# Addressing Diagnostic Challenges in Livedoid Vasculopathy: A Case Report on Interdisciplinary Management

**DOI:** 10.7759/cureus.72554

**Published:** 2024-10-28

**Authors:** Jose Ríos Padín, Delmarie M Rivera Rodríguez, Jose Nuñez Morales, Nelson Álvarez Cardín

**Affiliations:** 1 Internal Medicine, Veterans Affairs Caribbean Healthcare System, San Juan, PRI; 2 Internal Medicine, Universidad Central del Caribe School of Medicine, Bayamon, PRI

**Keywords:** chronic non-healing ulcers, cutaneous vasculature, dermal vessels, diagnostic workup, hypercoagulable state, livedoid vasculopathy, thrombotic occlusion

## Abstract

Livedoid vasculopathy (LV) is a rare vascular disorder characterized by excessive thrombosis of cutaneous vasculature, leading to dermal vessel occlusion, skin hypoxia, and ulceration. The nonspecific nature of its clinical manifestations often complicates diagnosis and inadequate oxygenation results in lesions that take longer to heal and are more susceptible to infections and complications. Despite its impact on patient quality of life, LV remains largely undocumented in the literature, making effective management challenging. This case report highlights the importance of a multidisciplinary approach in the diagnosis and treatment of LV. We detail the diagnostic process, including relevant laboratory findings and imaging studies, as well as therapeutic interventions tailored to the patient’s needs. The patient’s medical history, medication regimen, and laboratory workup are included to assist the medical community in establishing associations between existing comorbidities and LV. By sharing this case, we aim to raise awareness of LV and promote timely diagnosis and effective management strategies to improve patient outcomes.

## Introduction

The differential diagnosis for chronic, non-healing leg ulcerations in a patient with multiple comorbidities is broad and includes extensive workup. Evaluation of associated risk factors such as infection, vascular diseases, diabetes, nutrition, immunosuppressive therapy, immobilization, age, and social habits are important components that must be reassessed. Even afterward, there are multiple conditions with similar intercalating clinical presentations in which definite diagnoses rely on serum markers, imaging, and specific tissue biopsy. Livedoid vasculopathy (LV) is often overlooked as the underlying cause of these cases due to its varying presentation, lack of physician awareness, and rarity. LV is a rare disease characterized by chronic thrombotic occlusions of the dermal microvasculature [1]. While initially described as a type of vasculitis, it is now understood that LV does not result from vascular inflammation [2]. Instead, it involves cutaneous vascular damage caused by pauciinflammatory, and excessive thrombosis [2]. Previous literature has suggested a correlation of LV to coagulation disorders, rheumatologic diseases, and hypercoagulable states such as hemophilias, antiphospholipid syndrome, lupus, and malignancy [3].

LV lesions classically present bilaterally in the lower extremities with chronic, non-healing, recurrent ulcerations. Due to the nature of the vasculopathy, reduced blood flow occurs, and skin oxygen levels drop. These vascular events result in itching, painful bumps, and reddish-purple patches [4]. This condition progresses quickly to bleeding blisters or bullae, which can burst to form small, painful ulcers about 5 mm wide. These ulcers often merge into larger, painful lesions on the skin [4]. In this case report, we present a 78-year-old male patient with LV discovered upon admission for suspected osteomyelitis and describe the clinical presentation and workup toward diagnosis.

## Case presentation

A 78-year-old man presented to the emergency department with a complex medical history that included hypertension, type 2 diabetes mellitus, mixed hyperlipidemia, stage 2 chronic kidney disease, hypothyroidism, benign prostatic hyperplasia, and prostate cancer. His prostate cancer was treated with a radical prostatectomy in 2020, and metastatic adenocarcinoma was confirmed by iliac node biopsy in December 2023. The patient was referred from the hospital’s infectious diseases clinic for evaluation of multiple non-healing chronic ulcers associated with cellulitis in both lower extremities. His home medications included atorvastatin, enzalutamide, levothyroxine, lisinopril, and gabapentin.

Upon admission to the internal medicine ward, the patient was suspected of having a non-healing infected ulcer on the left leg, with cellulitis and a high suspicion of osteomyelitis. He reported that the lesions had begun approximately seven months prior after sustaining minor trauma from metal wires on a cyclone fence. Despite a variety of treatments, including oral antibiotics (clindamycin and cefuroxime), wound care, and a private holistic regimen that included endovenous laser therapy, hyperbaric oxygen sessions, and intravenous vitamin C, the ulcers continued to worsen. A culture taken two weeks prior had grown *Proteus mirabilis*, which was sensitive to levofloxacin; however, the patient was unable to tolerate this medication due to nausea. A recent consultation with a dermatologist had resulted in a punch biopsy being sent for pathology evaluation.

**Figure 1:**
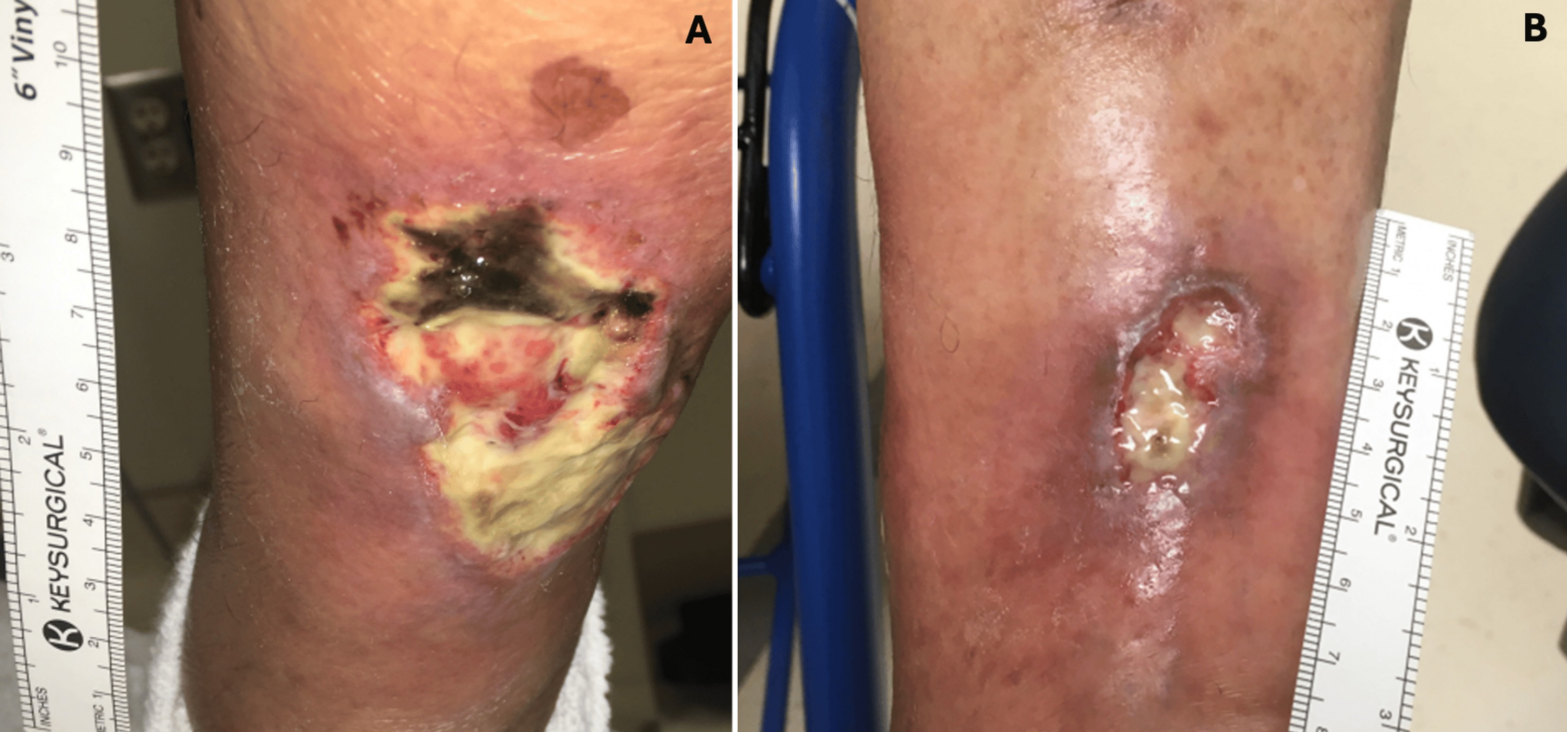
The patient's left extremity lesions at the time of admission A. The left lower extremity presented with hyperpigmentation and decreased hair distribution, accompanied by an erythematous, fibrinous, and edematous ulcer on the posterior leg measuring 7 cm x 5 cm x 0.5 cm. This ulcer exhibited purulent yellow secretions and necrosis at the upper edge. B. Additionally, a lesion on the anterior distal tibia measured 5 cm x 1.5 cm x 0.5 cm, characterized by fibrinous changes and surrounding erythema. Both ulcers were notable for tenderness and hyperalgesia, with associated itching and pain that worsened at night, despite the absence of objective fever.

**Figure 2:**
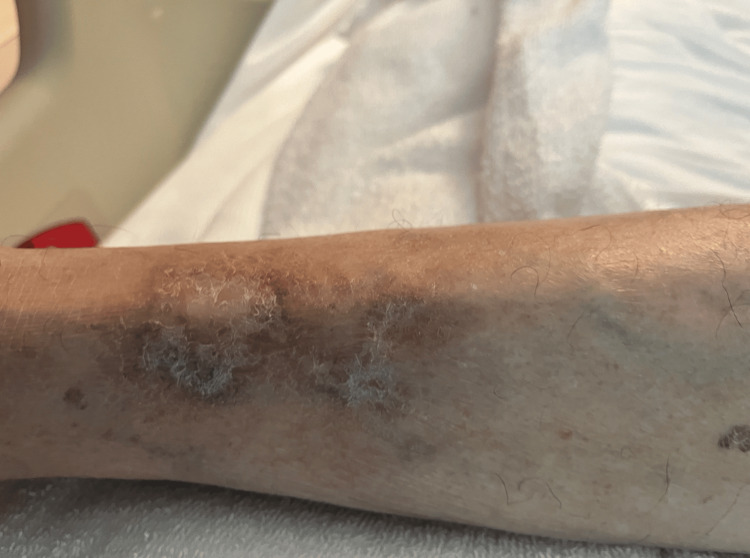
Atrophie blanche in patient's right extremity The right leg exhibits residual hyperpigmentation associated with previously healed ulcerations, a common observation in livedoid vasculopathy (LV). Surrounding the hyperpigmented areas are hypopigmented scar-like lesions with irregular borders, reflecting the skin's healing response to prior vascular injury and inflammation.

On physical examination, the lower left extremity exhibited hyperpigmentation with decreased hair distribution. There was an erythematous, fibrinous, and edematous posterior leg ulcer that demonstrated purulent yellow discharge and necrosis at the upper edge (Figure 1A). An anterior distal tibial ulcer was also noted, characterized by erythema and fibrinous changes (Figure 1B). Both ulcers were tender and exhibited hyperalgesia. The right leg displayed a scar-like, hypopigmented lesion from a previously healed ulcer (Figure 2).

The initial workup was directed toward ruling out osteomyelitis and evaluating factors contributing to poor wound healing, including potential vascular disease. Laboratory results revealed normocytic, normochromic anemia (Hgb=12.3 g/dL) without leukocytosis or thrombocytosis. Further workup for anemia revealed no abnormalities. Renal function, liver enzymes, and electrolytes were within normal limits. Inflammatory markers indicated a mildly elevated ESR of 43 mm/hr, while C-reactive protein (CRP) levels were normal. An ANA test was also positive at a titer of 1:160 with a speckled pattern, while complement levels (C3 and C4) remained within the normal range. A CT angiography could not be performed due to the patient's contrast allergy. Wound cultures subsequently grew* Pseudomonas aeruginosa*, which was initially treated with avibactam and ceftazidime before being switched to a 7-day IV course of cefepime.

A rheumatology consultation was requested due to the positive ANA and concerning pathology findings that raised the possibility of an underlying connective tissue disease. The hypercoagulable workup was negative for lupus anticoagulant, anticardiolipin, and β2-glycoprotein antibodies, but Protein C was elevated at 153%, potentially linked to the ongoing infection. The coagulation panel showed an INR of 1.32, PT of 16.7 seconds, and PTT of 33 seconds. Further workup was negative for antineutrophil cytoplasmic antibodies (ANCA), anti-dsDNA, and anti-extractable nuclear antigens (ENA) antibodies.

**Figure 3:**
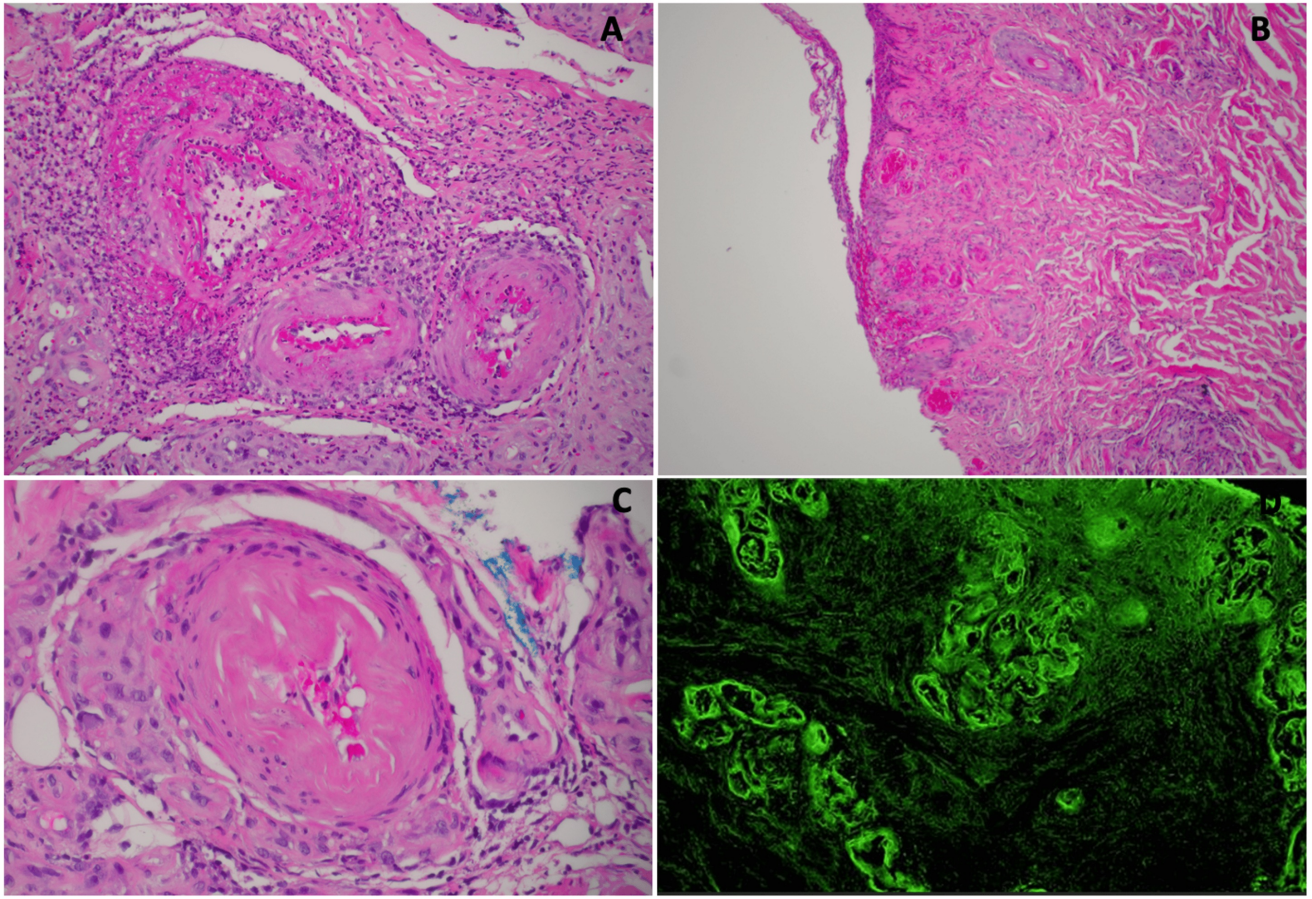
Histopathological and immunofluorescence findings derived from the lesion of the presented patient (A), (B), (C) The histopathological examination reveals a fragment of skin characterized by ulceration. The dermal and subcutaneous vessel walls are thickened, displaying significant infiltration by acute inflammatory cells. Additionally, fibrinoid necrosis is observed in the small and medium-sized vessels, indicating underlying vascular damage associated with the lesion. (D) A positive direct immunofluorescence test demonstrates perivascular fluorescence with fibrinogen deposition. Notably, all other tests (IgG, IgM, IgA, and C3) show negative fluorescence. The distinct pattern reveals homogeneous fibrinogen deposition on the thickened walls of the superficial and deep vascular plexus, accompanied by associated necrosis.

Shortly after admission, the dermatology punch biopsy revealed findings consistent with LV, including thickened dermal and subcutaneous vessel walls, fibrinoid necrosis, and positive immunofluorescence for fibrinogen (Figure 3). The management plan included the initiation of daily aspirin (81 mg) to address the risks associated with vasculopathy, along with coordinated follow-up care involving both dermatology and the patient’s primary care physician.

## Discussion

LV has an estimated incidence of one in 100,000, predominantly affecting women [5]. It primarily occurs in patients over 45 years of age, with peak incidence observed between ages 45-50 and 70-75 years [6]. Some studies indicate an average delay of approximately 6.65 years from the onset of initial symptoms to diagnosis, with a range spanning from 1 to 20 years [7]. This case diverges from typical LV trends, as the patient is male, over 75 years and received a relatively early diagnosis. However, it aligns with common trends in that LV patients frequently present with comorbidities, such as cardiovascular and renal diseases [5]. Arterial hypertension is the most frequent secondary diagnosis, followed by continuous therapy with anticoagulants and other medications [5]. Per these trends, our patient meets the criteria for such comorbidities.

LV is well-documented to be associated with hypercoagulable states, which may be autoimmune, hereditary, or malignant in nature [1]. The patient’s medical history is notable for autoimmune diseases, fatty liver, type 2 diabetes mellitus (T2DM), and prostate cancer with lymph node involvement, all of which have been reported to induce hypercoagulable states. Therefore, it is crucial to consider LV when a patient presents with chronic, non-healing ulcers in the context of multiple conditions that may induce a hypercoagulable state. Nonetheless, LV can occur independently or alongside various thrombophilic states and underlying systemic disorders. Aside from the patient's known comorbidities, positive antinuclear antibody (ANA), and Hexagonal Phase Lupus Anticoagulant test, no systemic presentations were identified.

Previous publications suggest that nonspecific individual and unique triggers can lead to the development of LV [1]. Initially described as livedoid reticularis with summer ulcerations, subsequent work indicates that increased temperatures may play a role in the etiology of LV [2,8]. Studies hypothesize that the presence of "pyroglobulins" or temperature-induced disturbances in coagulation could pave the way for LV [8]. The patient, in this case, attributes the development of his lesions to an injury sustained from the metal of a cyclone fence in August 2023, coinciding with the hottest August on record in Puerto Rico, according to the National Meteorology Service [9]. This critical aspect of the patient's clinical history supports the hypothesis that warm temperatures contribute to the pathogenesis and development of LV. Additionally, the patient's clinical history suggests that initial trauma, combined with elevated temperatures, created a favorable microenvironment for LV.

Although previous studies have reported marked improvement of LV with hyperbaric oxygen therapy, this treatment provided no resolution of ulceration or symptoms in our patient [10]. He received hyperbaric oxygen therapy two to three times a week and was scheduled to undergo a total of 26 sessions, along with a vitamin C serum and endovenous laser therapy. The patient did not complete the treatment, as his wounds worsened and expanded. He does not recall the exact number of sessions he completed; however, other studies indicate that hyperbaric oxygen therapy should occur five to six times a week for a minimum of 17 sessions [10]. Although data regarding treatment is limited, low-dose aspirin (81 mg) is considered the first-line antithrombotic therapy for patients without an identified thrombophilia. Antiplatelet therapy has been shown to be effective against the pathophysiology of LV, which further underscores the importance of this treatment approach [11-12].

The diagnostic process for LV requires clinicians to maintain a high degree of suspicion when patients present with lower extremity, non-healing, chronic ulcers. Such presentations could prompt a wide differential diagnosis ranging from osteomyelitis to venous ulcers, as these diagnoses are more familiar to clinicians. Therefore, it is imperative that clinicians adopt a multidisciplinary approach and initiate a comprehensive workup to exclude other diagnoses. Prior to admission, the patient’s primary care physician ordered a duplex ultrasound to rule out chronic venous insufficiency and peripheral arterial disease as potential underlying causes of the cutaneous lesions; the results were unremarkable. The enterostomal care team at the hospital suggested a referral to dermatology, suspecting calciphylaxis, which led to a biopsy being performed by the dermatology service. Despite this, the patient’s lesions were painful and itchy, prompting a visit to the infectious diseases outpatient clinic and, subsequently, a referral to the emergency room. Upon admission, the patient underwent extensive workup to rule out osteomyelitis and was empirically treated with antibiotics. The involvement of the rheumatology service was also crucial for excluding other autoimmune disorders that could account for the patient’s presentation. In total, seven services were involved in this case: primary care, enterostomal care, dermatology, infectious diseases, rheumatology, radiology, and pathology. The collaboration of these diverse services highlights the significance of a multidisciplinary approach in diagnosing LV. Furthermore, it underscores the necessity of clinician collaboration to diagnose rare clinical presentations, such as LV, and to ensure optimal patient care.

## Conclusions

In conclusion, LV poses diagnostic challenges due to its rarity, variable presentation, and lack of awareness among physicians. Our case report underscores the importance of a collaborative and multidisciplinary approach in the diagnosis and management of LV. Through detailed clinical presentation and workup, we aim to contribute to the medical community's understanding of LV and its association with various comorbidities. Prompt recognition and accurate diagnosis are essential to alleviate patient suffering and optimize outcomes in LV management. Further research and awareness are needed to enhance early detection and improve patient care in cases of LV.
